# Digitally Designed Semi-precision Attachment-Retained Cast Partial Dentures for Esthetics and Function: A Case Report

**DOI:** 10.7759/cureus.94511

**Published:** 2025-10-13

**Authors:** Mousumi Mahato, Sadananda Hota, Amulya Jain, Jaya Naikode

**Affiliations:** 1 Prosthodontics, Kalinga Institute of Medical Sciences, Bhubaneswar, IND; 2 Prosthodontics, Kalinga Institute of Dental Sciences, Bhubaneswar, IND; 3 Prosthodontics and Crown and Bridge, Kalinga Institute of Dental Sciences, Bhubaneswar, IND; 4 Periodontology, KM Shah Dental College, Vadodara, IND

**Keywords:** custom attachment, digital prosthodontics, removable partial denture, semi-precision attachment, vertical dimension

## Abstract

Removable partial dentures (RPDs) retained by semi-precision attachments are valuable alternatives to conventional clasp-retained prostheses, particularly in cases where esthetics and biomechanical stability are critical. With advancements in digital dentistry, customized attachments and frameworks can now be fabricated with high accuracy and predictability. This case report describes the prosthetic rehabilitation of a 66-year-old male patient with Kennedy Class II mod 1 maxilla and Kennedy Class I mandible using digitally fabricated crowns and a semi-precision attachment-retained RPD. A custom extracoronal patrix was incorporated on tooth 11, eliminating metal display and enhancing esthetics. A 3 mm increase in vertical dimension was achieved, and the patient has been followed up for two years without complications. This report underscores the role of digital technology in delivering esthetically driven and functionally sound outcomes in partially edentulous cases.

## Introduction

Prosthodontic rehabilitation of partially edentulous patients requires careful consideration of function, esthetics, and patient-specific constraints. While fixed partial dentures and implant-supported prostheses are preferred for their longevity and comfort, they are not always feasible, particularly in medically compromised or surgically averse patients [1]. Removable partial dentures with semi-precision or precision attachments offer a conservative and esthetically superior solution compared to traditional clasp-retained dentures. Precision attachments are prefabricated, milled metal components with extremely tight tolerances for a rigid, metal-on-metal connection, often intracoronal, while semi-precision attachments are custom-fabricated from wax, plastic, or nylon patterns, have looser tolerances, may contain flexible (resilient) components, are typically extracoronal, and are less costly.

Recent advances in digital technology have allowed clinicians to design and fabricate crowns, attachments, and removable partial denture (RPD) frameworks with unprecedented accuracy. Custom extracoronal attachments, digitally designed and fabricated, can significantly enhance the esthetic and functional outcomes of RPDs. This report presents the use of a digitally fabricated semi-precision attachment to restore esthetics and function in a patient unwilling to undergo implant surgery. Use of a digitally designed custom extracoronal patrix on a maxillary central incisor integrated into a direct metal laser sintering (DMLS) framework improves the novelty of this case [2].

## Case presentation

**Figure 1:**
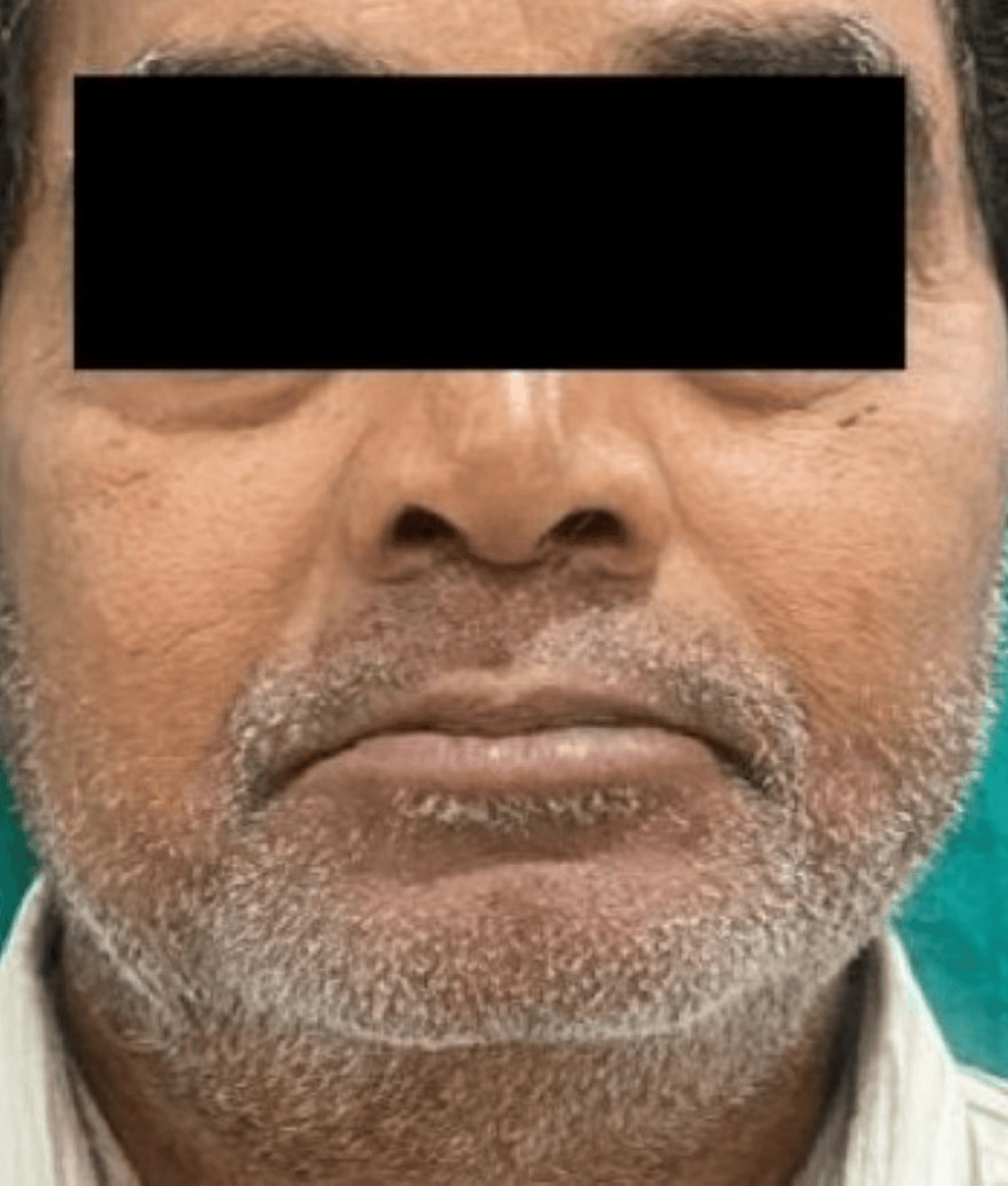
Pre-operative extraoral frontal view

**Figure 2:**
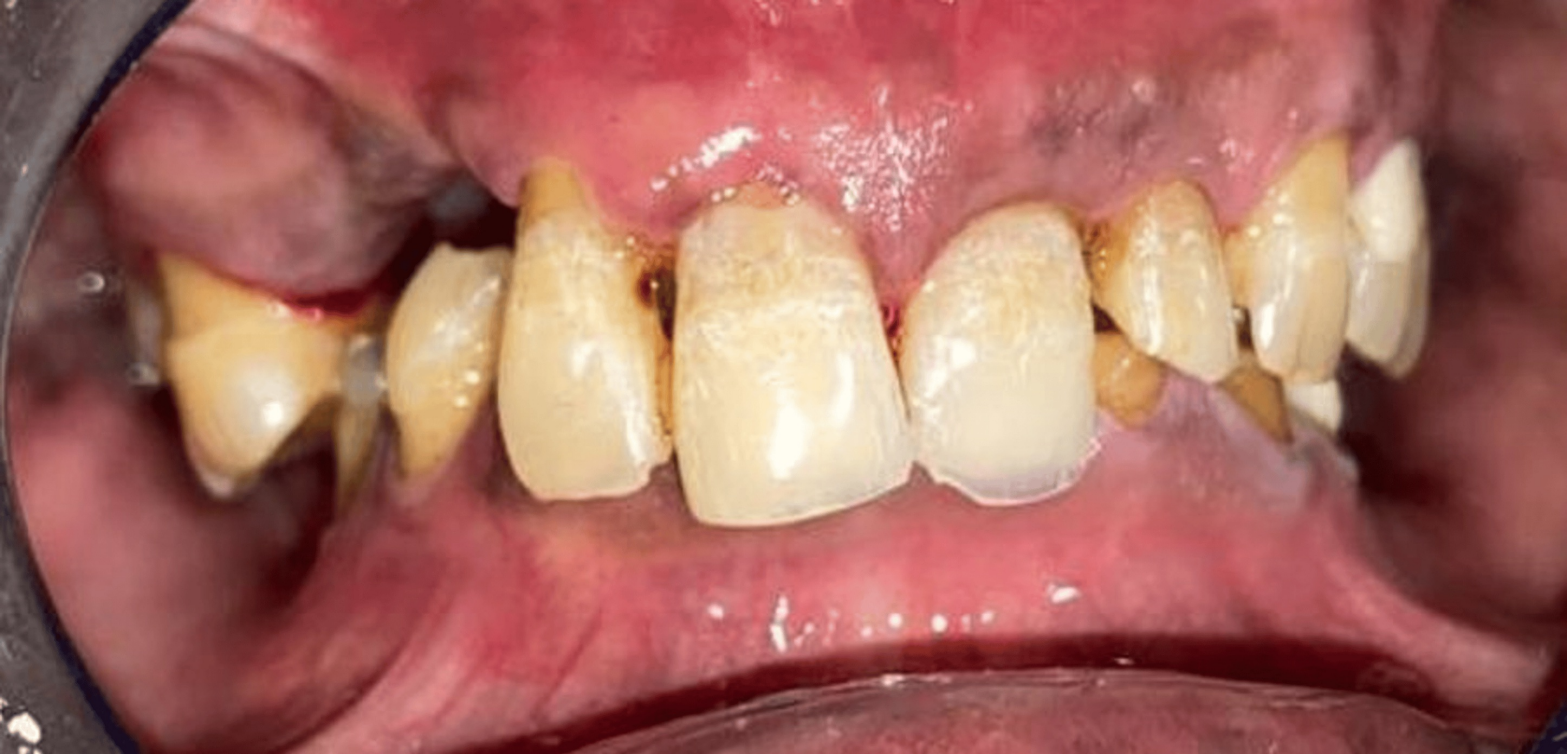
Pre-operative intraoral frontal view (maximum intercuspation)

A 66-year-old man reported to the Department of Prosthodontics and Crown & Bridge with chief complaints of difficulty in chewing and an unesthetic appearance, seeking rehabilitation of multiple missing teeth. Clinical and radiographic evaluation revealed missing teeth: 12, 13, 14, 15, 26, 27, 36, 37, 46, and 47. Kennedy's class 2 MOD 1 in the maxilla and Kennedy's class 1 in the mandible [3]. He presented with loss of posterior support and reduced vertical dimension of occlusion (VDO), also evident extraorally with signs like drooping corners of the mouth (Figure 1), along with mild supraeruption in the opposing arches. Tooth 12 was advised for extraction due to mobility. The remaining residual ridge was high well rounded in the maxilla (Class III) and low well rounded in the mandible (Class V) (Cawood and Howel classification) [4]. Teeth 16 and 17 were also advised for extraction due to poor periodontal health (Figure 2).

The patient had no significant financial concerns but refused implant therapy due to a strong family history of diabetes and a personal aversion to surgery. A fixed bridge was ruled out due to a long edentulous span in the maxilla and bilateral distal extension in the mandible. A removable prosthesis with a semi-precision attachment was considered most suitable as the patient was conscious about metal display due to the clasp in the incisor region.

Treatment plan

The definitive treatment plan included: metal-ceramic splinted DMLS (direct metal laser sintering) crowns in the maxillary and mandibular arches and a custom semi-precision patrix attachment on tooth 11, followed by a digitally designed cast partial denture (CPD) with integrated rest seats in both arches. There was an increased vertical dimension of 3 mm.

Clinical procedure

**Figure 3:**
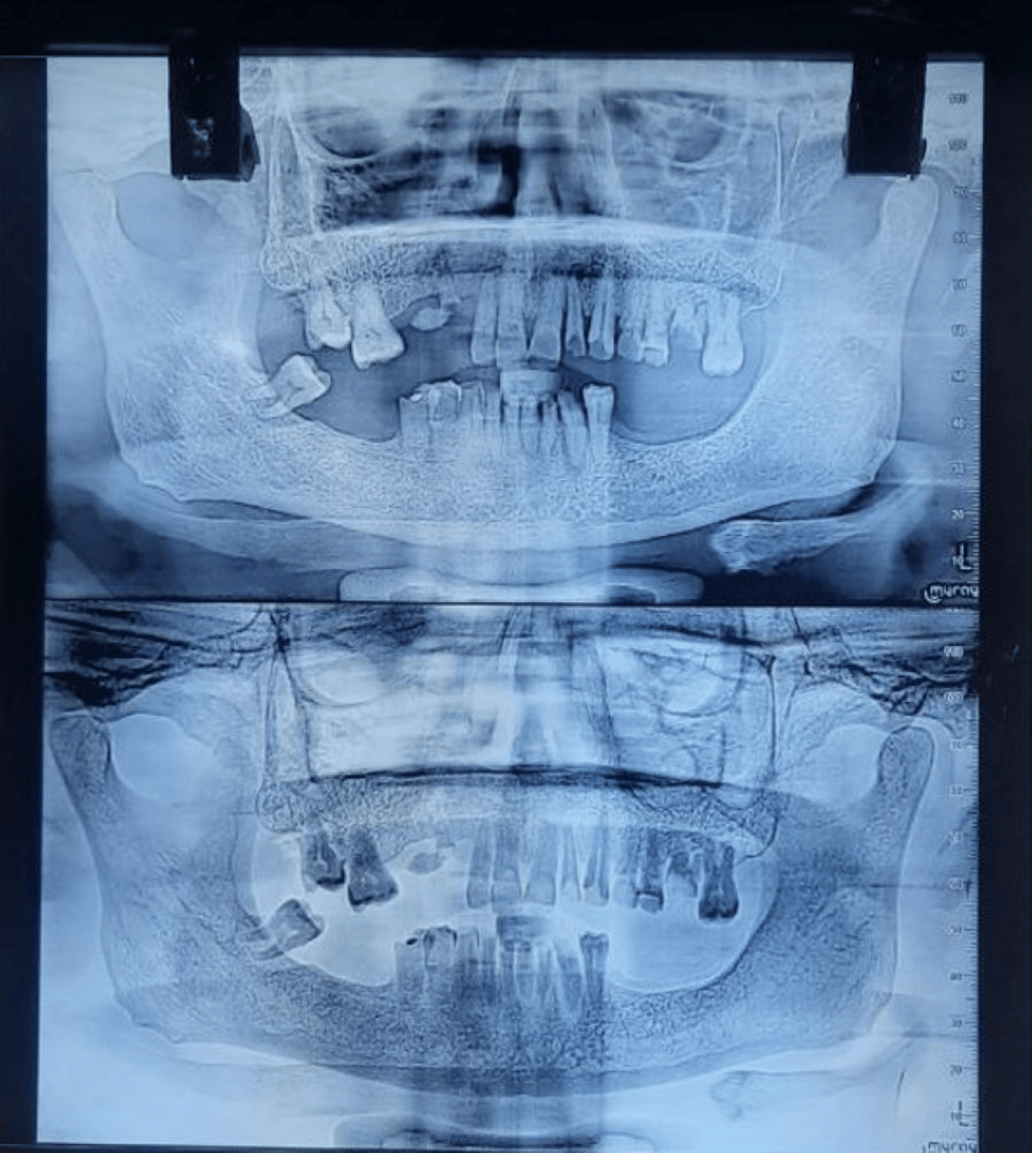
Preoperative orthopantomogram (OPG) Based on the OPG, teeth number 12, 15, 48, 26, and 27 were planned for extraction due to poor periodontal health, and the remaining were planned for endodontic treatment due to attritional pulp exposure and sensitivity.

**Figure 4:**
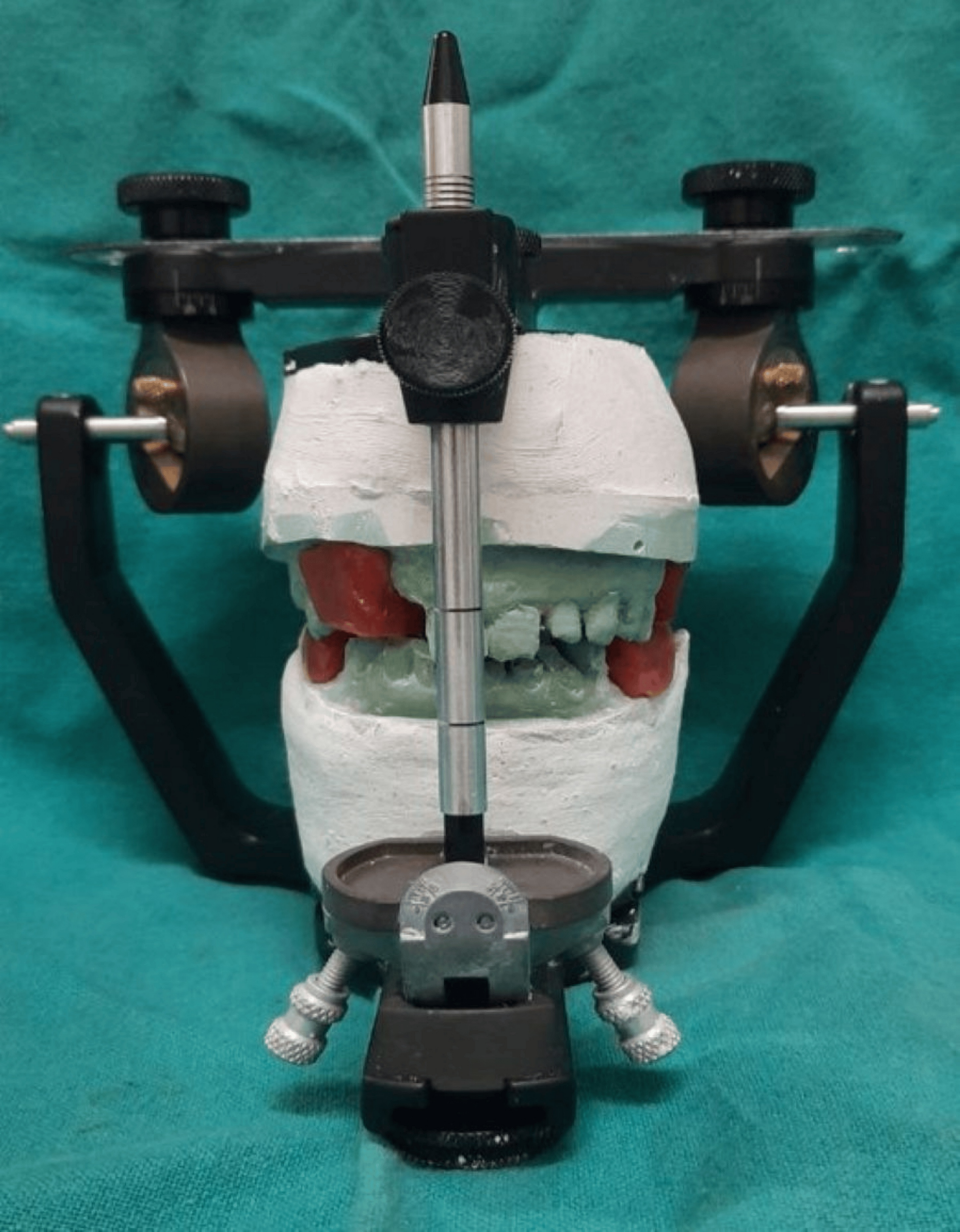
Mounting of casts to a semi-adjustable articulator with the help of facebow apparatus

Preoperative orthopantomogram (OPG) (Figure 3) was obtained prior to extraction planning to assess the existing dentition and residual ridges. Preliminary impressions were made, and diagnostic casts were mounted on a semi-adjustable articulator (Hanau Wide Vue Articulator) using a facebow transfer to accurately replicate the patient’s maxillomandibular relationship (Figure 4). The vertical dimension was found to be reduced, and a planned increase of 3 mm was verified using interim prostheses over a trial period to ensure patient adaptability and comfort.

**Figure 5:**
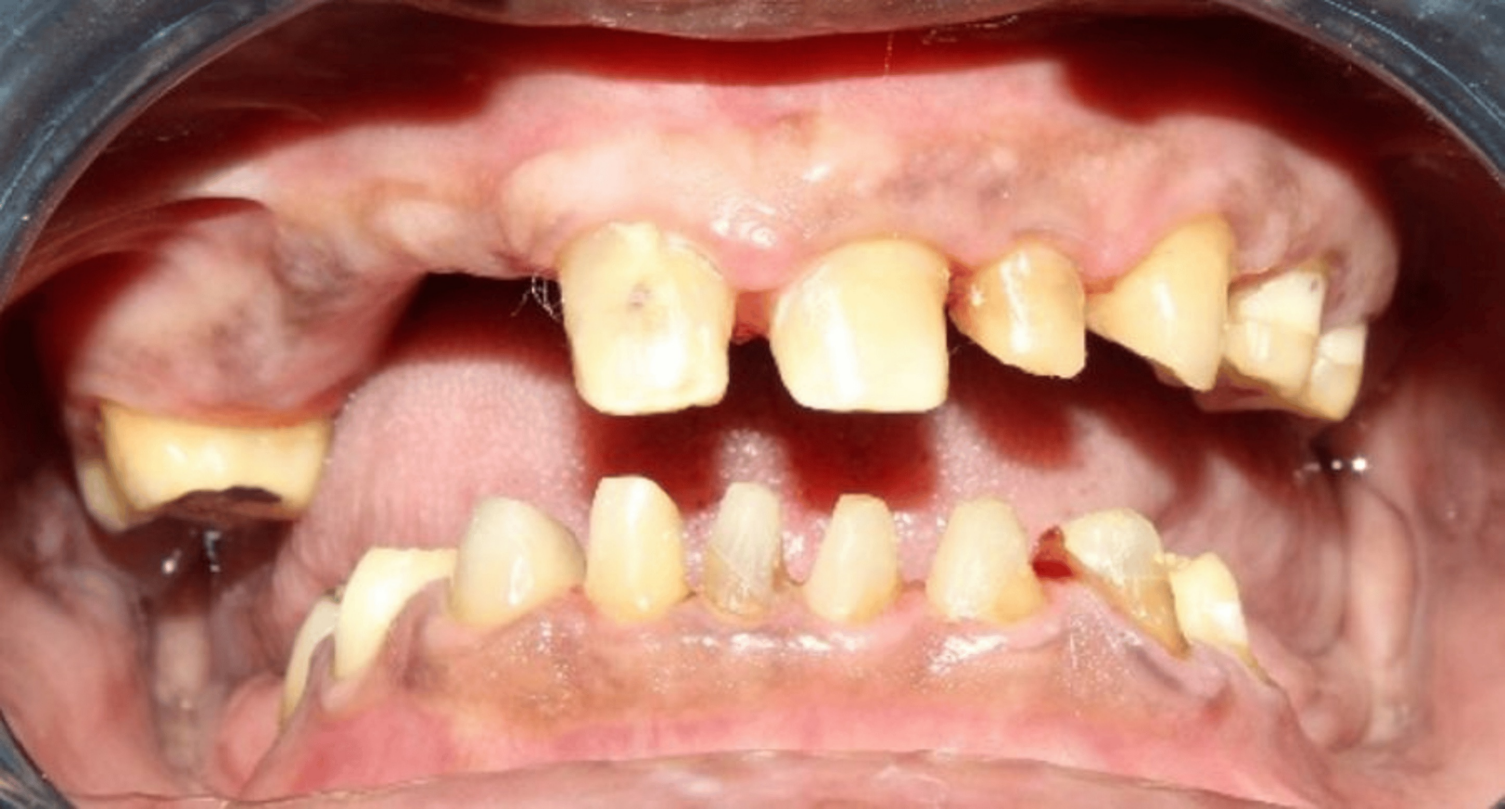
Tooth preparation for DMLS crowns DMLS: Direct metal laser sintering

**Figure 6:**
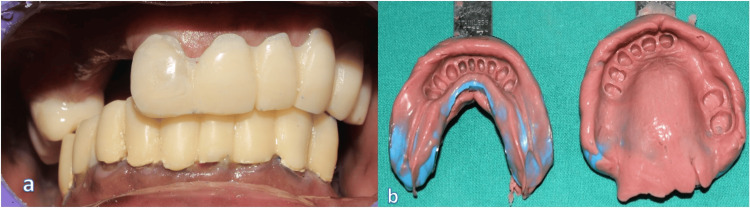
Temporary crowns according to the increased vertical dimension and final impressions in heavy-body and light-body elastomeric impression materials a. Temporary crowns according to increased vertical dimension b. Final impressions - mandible and maxilla

**Figure 7:**
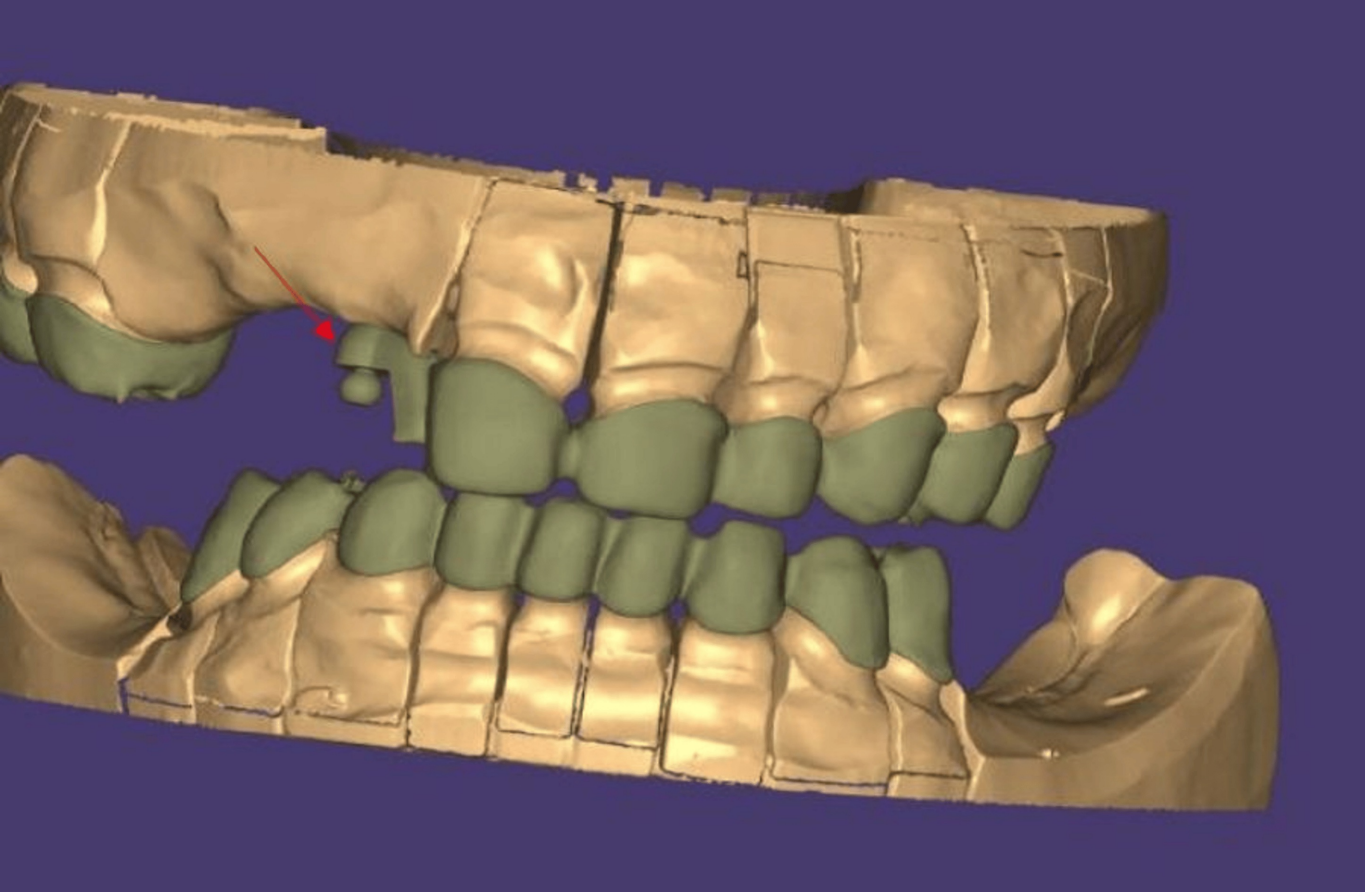
Digital designing of crowns and patrix component The arrow points to the patrix component

Following this, teeth 11 to 25 in the maxilla and the remaining mandibular teeth were prepared following fiber post and core, where full-coverage metal-ceramic DMLS restorations were required, followed by impression making using an irreversible hydrocolloid impression material for preparation of cast for temporization (Figure 5). Temporization was performed at the increased vertical dimension using auto-polymerizing acrylic resin, allowing for function and esthetics during the healing and adaptation phase (Figure 6), followed by final impression making using a heavy- and light-body addition silicone elastomeric material. Crowns were digitally designed using Exocad CAD software, enabling precise control over contours, contacts, and prosthetic space allocation for the semi-precision attachment. The copings were fabricated using Direct Metal Laser Sintering (DMLS) technology along with the design for the patrix distal to the 11 region (Figure 7).

**Figure 8:**
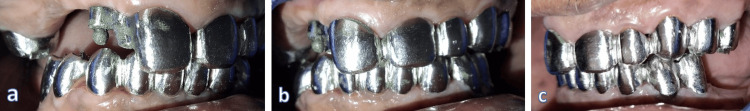
Trial for metal copings a. Right lateral view; b. Frontal view; c. Left lateral view

A custom-designed extracoronal patrix was digitally integrated onto the distal aspect of tooth 11, ensuring optimal positioning and parallelism with the prosthesis path of insertion. Crowns from 11 to 25 and posterior crowns on 16 and 17 were splinted to enhance prosthetic stability and distribute functional forces. The mandibular abutment crowns were similarly splinted. The remaining seats were strategically incorporated in 11, 16, 24, 25, 34, 35, 44, and 45 to provide vertical support and prevent tissue-ward movement of the removable prosthesis during function. A metal trial was performed to verify marginal integrity and passivity of the substructure (Figure 8).

**Figure 9:**
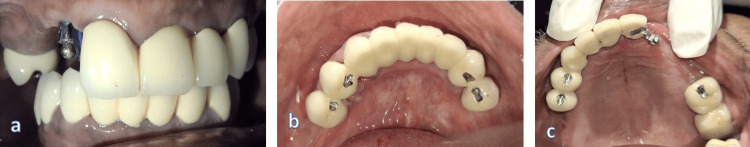
Final cementation of maxillary and mandibular DMLS crowns a. Frontal view; b. Mandibular occlusal view; c. Maxillary occlusal view DMLS: Direct metal laser sintering

Final ceramic layering was finished for best esthetics, followed by a trial of bisque to assess occlusion, shade, and intraoral harmony (Figure 9). After confirmation, cementation of all crowns was done with resin-modified glass ionomer cement, selected for its high fluoride release, chemical bonding to tooth structure, ease of handling, and optimal film thickness for ensuring the proper seating of multiple units. After cementation, final impressions were recorded with additional silicone impression material to accurately capture the fixed elements.

**Figure 10:**
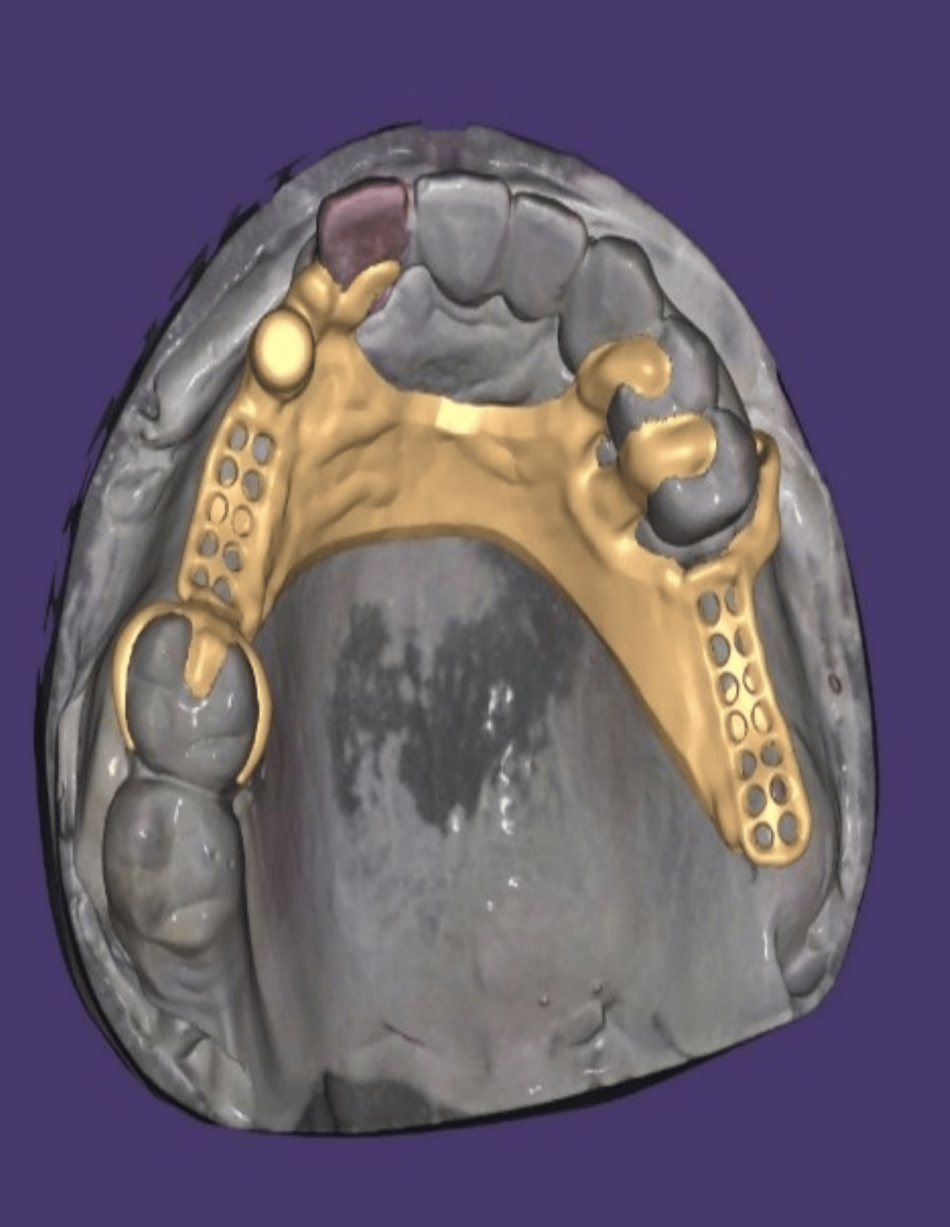
Exocad file of upper CPD design with semi-precision attachment CPD: Cast partial denture

**Figure 11:**
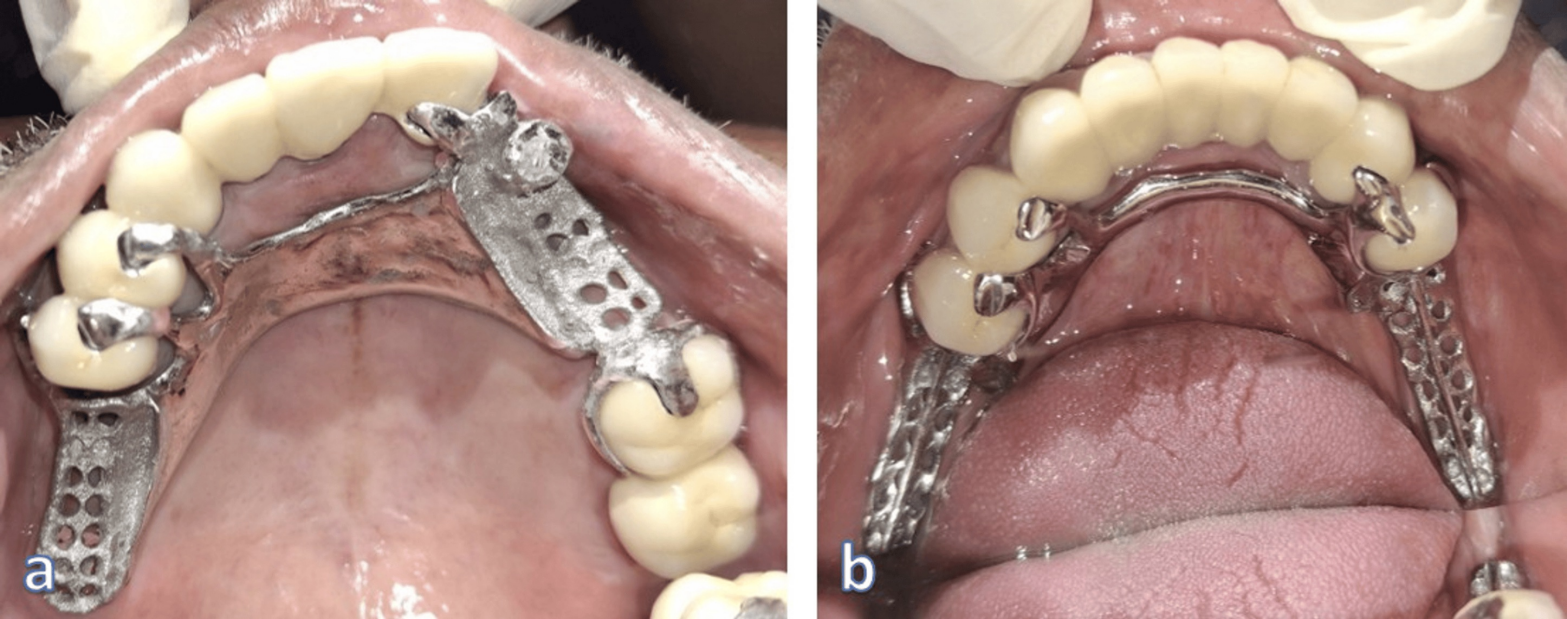
Trial of the cast partial denture framework a. Maxilla; b. Mandible

The CPD frameworks were digitally designed with rest seats, connectors, and direct and indirect retainers where required, and semi-precision attachment was planned in the position to avoid designing of I-bar in the incisor region thereby providing esthetics with function followed by computer-aided milling to provide a passive fit and integration with the pre-positioned attachment (Figure 10). At the framework trial, intraoral assessment revealed superior adaptation and correct seating of the female attachment component (Figure 11).

**Figure 12:**
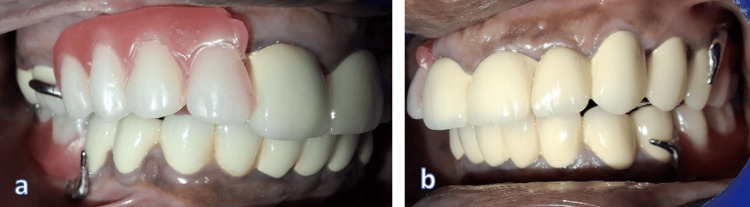
Wax try-in of cast partial dentures a. Right lateral; b. Left lateral

**Figure 13:**
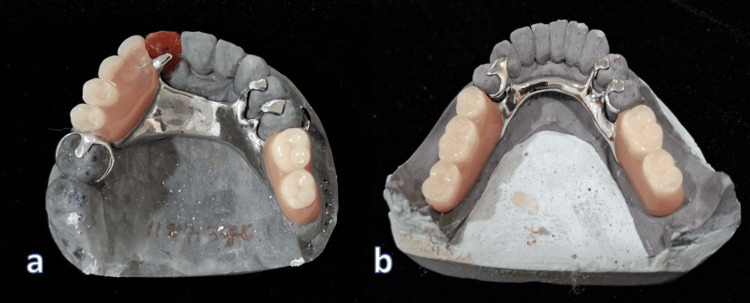
Acrylization of upper and lower cast partial dentures a. Upper cast partial denture; b. Lower cast partial denture

Occlusal registration was captured with wax rims on the frameworks, and the teeth were positioned in wax to replicate natural dentition. Trial insertion (Wax try-in) was carried out to confirm occlusal contacts, esthetics, and phonetics, with slight refinements based on patient feedback (Figure 12). The CPDs were then acrylized by using heat-cure acrylic resin to provide strength and color stability (Figure 13).

**Figure 14:**
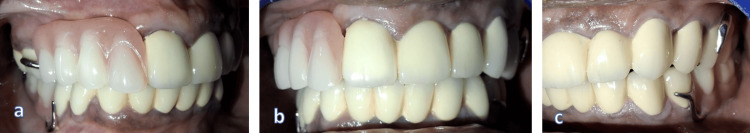
Final insertion of cast partial dentures a. Right lateral view; b. Frontal view; c. Left lateral view

**Figure 15:**
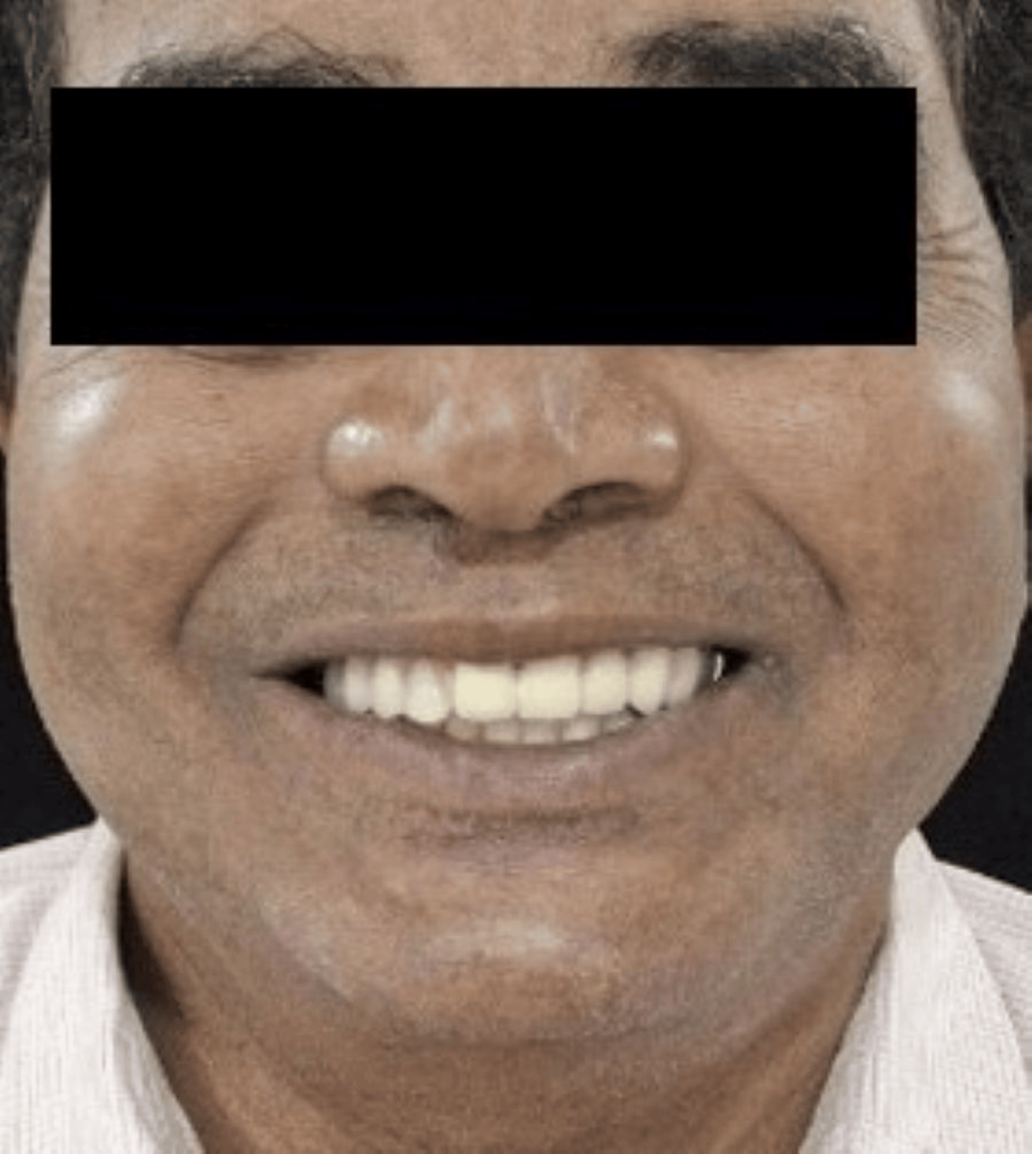
Post-operative extraoral frontal view

During final insertion, the prostheses had good retention, esthetics, and stability. Post-insertion instruction was given, noting oral hygiene, prosthesis maintenance, and recall intervals (Figure 14). The patient was followed up at one month, six months, one year, and two years. Throughout this period, no complications, biological or mechanical, were observed. The prostheses remained functionally stable, and the patient expressed high satisfaction with both appearance and comfort (Figure 15). During follow-up visits, the patient reported lesser trouble in pronouncing words, lesser discomfort during chewing food, and improved functions as well as improved social confidence due to better dental and facial appearance [5].

## Discussion

Removable prosthodontics continues to play a critical role in the rehabilitation of partially edentulous patients, especially when implants are contraindicated. In such situations, RPDs with precision or semi-precision attachments offer improved retention, esthetics, and patient acceptance [6].

In this case, the use of a digitally designed extracoronal patrix on tooth 11 allowed for esthetic anterior rehabilitation without the display of metal clasps. The attachment's location was chosen carefully to maintain biomechanical principles, and adjacent teeth were splinted to distribute functional loads. Incorporating rest seats on strategically selected abutments ensured support and stability for the RPD.

The 3 mm increase in vertical dimension was well-tolerated, highlighting the importance of phased adaptation using interim prostheses. Furthermore, digital technology provided enhanced control over design parameters, enabling parallelism of attachments, precise crown contours, and accurate framework fabrication.

Multiple studies have highlighted the esthetic advantages and functional success of extracoronal attachments, particularly when clasping in the anterior segment would be visible [7-9]. Moreover, digital workflows are now widely recognized for improving prosthesis fit and reducing clinical time [10,11].

Limitations

Results from a single patient cannot be generalized, and longer-term comparative studies are needed to validate the superiority of digitally fabricated semi-precision attachments over conventional designs. Factors such as laboratory expertise, cost, and accessibility may influence the replicability of such results in different clinical settings.

## Conclusions

This case demonstrates that a digitally designed semi-precision attachment-retained RPD can be a successful and esthetically acceptable treatment option in patients unwilling to undergo implant therapy. The integration of digital design and additive manufacturing technologies allowed precise control over prosthetic contours, attachment placement, and occlusion. A two-year follow-up showed high patient satisfaction and prosthetic stability, reinforcing the long-term viability of digitally fabricated semi-precision RPDs in contemporary prosthodontic practice.
